# A Two-Gene Blood Test for Methylated DNA Sensitive for Colorectal Cancer

**DOI:** 10.1371/journal.pone.0125041

**Published:** 2015-04-30

**Authors:** Susanne K. Pedersen, Rohan T. Baker, Aidan McEvoy, David H. Murray, Melissa Thomas, Peter L. Molloy, Sue Mitchell, Trevor Lockett, Graeme P. Young, Lawrence C. LaPointe

**Affiliations:** 1 Clinical Genomics Proprietary Limited, Sydney, Australia; 2 Food & Nutrition Flagship, Commonwealth Scientific and Industrial Research Organisation, Sydney, Australia; 3 Flinders Centre for Innovation in Cancer, Flinders University of South Australia, Adelaide, Australia; Pontificia Universidad Catolica de Chile, Faculty of Medicine, CHILE

## Abstract

**Background:**

Specific genes are methylated with high frequency in colorectal neoplasia, and may leak into blood. Detection of multiple methylated DNA biomarkers in blood may improve assay sensitivity for colorectal cancer (CRC) relative to a single marker. We undertook a case-control study evaluating the presence of two methylation DNA markers, *BCAT1* and *IKZF1*, in circulation to determine if they were complementary for detection of CRC.

**Methods:**

Methylation-specific PCR assays were developed to measure the level of methylated *BCAT1* and *IKZF1* in DNA extracted from plasma obtained from colonoscopy-confirmed 144 healthy controls and 74 CRC cases.

**Results:**

DNA yields ranged from 2 to 730 ng/mL plasma (mean 18.6ng/mL; 95% CI 11-26 ng/mL) and did not correlate with gender, age or CRC status. Methylated *BCAT1* and *IKZF1* DNA were detected in respectively 48 (65%) and 50 (68%) of the 74 cancers. In contrast, only 5 (4%) and 7 (5%) controls were positive for *BCAT1* and *IKZF1* DNA methylation, respectively. A two-gene classifier model (“either or” rule) improved segregation of CRC from controls, with 57 of 74 cancers (77%) compared to only 11 of 144 (7.6%) controls being positive for *BCAT1* and/or *IKZF1* DNA methylation. Increasing levels of methylated DNA were observed as CRC stage progressed.

**Conclusions:**

Detection of methylated *BCAT1* and/or *IKZF1* DNA in plasma may have clinical application as a novel blood test for CRC. Combining the results from the two methylation-specific PCR assays improved CRC detection with minimal change in specificity. Further validation of this two-gene blood test with a view to application in screening is now indicated.

## Introduction

Colorectal cancer (CRC) is a major public health challenge and screening is an important tool in cancer control in countries with a significant CRC burden. However, participation rates with screening based on invasive colonoscopy or fecal sampling are low [1]. A recent survey of screening-age subjects supports the likely acceptability of a blood test for CRC with 78% of the participants preferring a blood test to a stool test [2].

Recent improvements in molecular technologies have demonstrated that tumour-derived nucleic acids in blood can be detected in a robust and reproducible manner [3]. In particular, there is accumulating literature documenting circulating tumour-specific mutated and/or methylated DNA and RNA [4–8].

We have previously described the discovery and validation of a range of novel hypermethylated genes characteristic of colorectal neoplastic tissue [9]. Methylation specific assays designed to detect these hypermethylated genes showed minimal signal in DNA extracted from pooled blood of healthy donors for a subset of the markers [9]. Based on their relative levels of potential background we chose two genes, branched-chain amino acid transaminase 1, *BCAT1*, and ikaros family zinc finger protein 1, *IKZF1* [9,10] as lead candidates for evaluation as the basis of a blood test for CRC.

We report the evaluation of two methylation-specific PCR assays for detection of methylated *BCAT1* and *IKZF1* DNA extracted from plasma samples from 218 colonoscopy-examined individuals (144 healthy controls and 74 CRC cases) to determine if the methylation biomarkers were complementary for detection of CRC.

## Materials and Methods

### Cases and controls

Blood was obtained from volunteering and informed subjects scheduled for colonoscopy at Flinders Medical Centre (FMC) or Repatriation General Hospital (Adelaide, SA, Australia) who gave written consent in concordance with the study protocol reviewed and approved by the Research and Ethics Committee of the Repatriation General Hospital and Ethics Committee of Flinders Medical Centre. Blood was collected by phlebotomy after bowel preparation but prior to colonoscopy. Clinical diagnosis was determined on the basis of colonoscopic findings and histological assessment. Cancers were staged according to AJCC Cancer staging 7^th^ edition. Additional plasma specimens from colonoscopy-examined subjects were sourced from Proteogenex Inc. CA, USA (PGX) who collected blood from volunteers 3–10 days after their exploratory colonoscopy. Specimens were selected for analysis retrospectively to colonoscopy-based diagnosis. Cancer cases (FMC: n = 25, PGX: n = 49) was selected based on volume availability, whereas controls (FMC: n = 85, PGX: n = 84) were selected based on no appearance of hyperplastic and adenomatous polyps (but allowing benign conditions such as hemorrhoids and diverticular disease), with an attempt to gender-age match the CRC cases available for testing.

### Plasma preparation

The plasma component from whole blood collected in K_3_EDTA Vacuette tubes (Greiner Bio-One, Frickenhausen, Germany) was isolated by centrifugation at 1,500g for 10 minutes at 4°C (brakes disabled on centrifuge). The top-layer plasma fraction was retrieved and centrifugation was repeated. The resulting ‘two-spin’ plasma was stored at -80°C until further use. The total time from blood draw to freezing of plasma was no more than four hours at all recruitment sites.

### Process Controls

Pooled plasma from gender- and age-matched donors under 30 years of age was commercially sourced (Bioreclamation, NY, USA) and used neat as a negative control or spiked with sonicated fully methylated human genomic DNA (Millipore, MA, US), 1250pg/mL, as a positive control. These process controls were made as 2 litre batches and subsequently stored as 4mL aliquots at -80°C until further use.

### Study Design

Plasma specimens were randomly processed in batches of 24 samples including one positive process control, one negative process control and 22 clinical samples (phenotypes blinded to assay operators).

### Isolation and bisulphite conversion of cell-free circulating DNA

Cell-free circulating DNA (cfDNA) was extracted from 4mL plasma using the QIAamp Circulating Nucleic Acid Kit (Qiagen, Hilden, Germany). The extraction was semi-automated on two QIACube liquid handlers (12 samples per instrument per run) as recommended by manufacturer (Qiagen). Elution volume was set to 40μL and the eluate was manually re-applied to the silica column to improve cfDNA yield. The EpiTect Fast Bisulfite Conversion kit (Qiagen) was used to bisulphite convert isolated cfDNA. The bisulphite converted DNA was purified on QIACube instrumentation using the EpiTect Plus Bisulfite kit (Qiagen) with two modifications: 1) columns from the QIAamp Circulating Nucleic Acid kit were used in place of columns provided in the Epitect Plus Bisulfite kit; 2) The Epitect Plus Binding buffer was prepared with isopropanol in place of ethanol. Elution volume was set to 40μL and the eluate was manually re-applied to the silica column to improve cfDNA yield. A total of 36μL bisulphite converted cfDNA was recovered from each 4mL plasma specimen. Successful recovery of bisulphite converted cfDNA was confirmed on duplicate input of 5μL 1:5 diluted bisulphite converted DNA using an ACTB PCR assay as previously described [11] with minor modifications (see S1 Table). Each PCR run included three no-template control samples and a standard curve based on a 4-fold serial dilution of 5ng bisulphite-converted human DNA isolated from white blood cells (WBC; Roche Applied Science, Basel, Switzerland) prepared in a background of nuclease-free water (Promega, WI, USA). The mass of recovered bisulphite converted cfDNA for each processed specimen was estimated from the standard curve using linear regression fits to Cycle threshold (Ct) values (see S1 Fig).

### Detection of methylated *BCAT1* and *IKZF1* DNA

Appearance of methylated *BCAT1* and *IKZF1* DNA in circulation was measured on triplicate input of 5μL purified bisulphite converted DNA (e.g. equivalent to 0.5mL plasma per PCR) using bisulphite conversion- and methylation specific assays as previously described [9] with minor modifications (see S1 Table). Briefly, the two real-time PCR assays targeted methylated CpG sites in regions spanning 102- or 95- nucleotides, either within, or just upstream of the first exon of the *BCAT1* and *IKZF1* genes, respectively (see S2 Fig). DNA target amplification was performed for 50 cycles in an LC480 lightcycler, Model II (Roche). Ct values were calculated using a 2^nd^ derivative algorithm provided with the LC480 software. The *BCAT1* PCR assay (hydrolysis probe) and *IKZF1* PCR assay (SYBR green/melt) were qualitatively called “positive” for *BCAT1* methylation if a total change in fluorescence intensity above background levels was measured within 50 PCR amplification cycles. Further, for *IKZF1* positive callings, the melt profile analysis component of the *IKZF1* PCR assay had to give a melting temperature greater than 80°C (see S1 Table). PCR assays with no signal were assigned an arbitrary Ct value of 50 for graphic purposes. Each PCR run included three no-template control samples, three 5ng bis-converted WBC DNA (Roche) negative control samples, and a standard curve based on 4-fold serial dilution of 5ng bisulphite-converted fully methylated human genomic DNA (Millipore) prepared in a background of bisulphite-converted WBC DNA (Roche) (see S2 Fig). Each standard point concentration contained a total of 5ng DNA. The methylation specific assays were quantitative from 20pg (0.4%) to 5ng (100%) of methylated target DNA in a background of WBC DNA (R^2^ square values: *BCAT1*, 0.9370; *IKZF1*, 0.9387, S2 Fig), but able to detect down to 5pg (equivalent to 1–2 genomic copies) of methylated *BCAT1* and *IKZF1* DNA. The mass of methylated *BCAT1* and *IZKF1* DNA for each processed specimen was estimated from the standard curve using linear regression fits to Ct values. The mass of methylated *BCAT1* or *IKZF1* DNA was expressed as the average mass (picogram; pg) of methylated *BCAT1* or *IKZF1* DNA per triplicate PCR assay or per mL plasma (see S2 Table).

### Sample Result Interpretation

Recovery of bisulphite converted DNA was confirmed if at least one replicate was positive in the *ACTB* qPCR assay. The remaining bisulphite converted DNA was thereafter analysed as triplicate inputs of 5μL in either the *BCAT1* or *IKZF1* qPCR assays. The following data were collected for each processed sample: two *ACTB* qPCR Ct measurements, mass of bisulphite converted DNA, three *BCAT1* qPCR Ct measurements, three methylated *BCAT1* mass measurements, three *IKZF1* qPCR Ct measurements and three methylated *IKZF1* measurements (S2 Table). All PCR amplification curves were visually inspected. Before unmasking sample phenotypes, a specimen was qualitatively defined as clinically positive for CRC reporting purposes if any of the triplicates in the *BCAT1* or *IKZF1* PCR assays were positive.

### Statistical analysis

Statistical analyses were performed using GraphPad Prism v5.0d for Mac OS X (GraphPad Software, San Diego, CA). For density plots of amounts of methylated *BCAT1* or *IKZF1* DNA, samples with “no signal” results were omitted. Each of the triplicate assays contained DNA isolated from the equivalent of 0.5mL plasma. The empirical density of ln(methylated *BCAT1*, pg) or ln(methylated *IKZF1*, pg*)* was estimated separately for healthy controls, early stage (Stage I+II) and late stage (Stage III+IV) cancer. Assuming a Gaussian distribution for the methylation mass values (pg), to give the maximum entropy (the worst case) with the observed average and variance, the densities were again estimated. Using these densities, we calculated the cumulative distribution function (F(x) = P(X≤x)) and report 1-F(x) for ln(pg) methylated *BCAT1* and *IKZF1* values of 3, 3.9 and 4.6. We also report the likelihood ratio relative to healthy controls.

## Results

### Isolation of cell-free DNA from plasma specimens

Ten individual batches were performed to process the 218 plasma samples. Process Controls confirmed no confounding variation in batch processing (data not shown). The *ACTB* qPCR assay confirmed successful recovery of bisulphite converted DNA from all 218 processed plasma specimens (see S3 Fig and S2 Table) and the median DNA concentrations relative to phenotype and clinical conditions of collection are summarized in Table 1. The majority of the recovered DNA yields were within a two-fold concentration range (95%CI range: 11.2–26.2ng/mL). Significantly lower yields of bisulphite converted DNA were recovered from specimens collected by FMC compared to the average yields recovered from specimens collected by PGX (median concentrations: FMC: 7.0 ng/mL, PGX: 17.8ng/mL, t-test p value <0.0001, see S3 Fig). Inter-site protocol differences have been reported to cause the strongest variation in measured circulating DNA concentrations [12]. We carefully monitored collection and processing procedures to ensure common protocols. The only systematic variation that might account for these contrasting DNA yields was the different time points at which blood was collected relative to colonoscopy. There was no correlation between amount of DNA recovered from plasma and clinical phenotype (see S3 Fig) in contrast to studies that included a higher proportion of late stage cancers [13,14]. Furthermore, no correlation was observed between DNA yield, patient age or gender (data not shown).

**Table 1:** Measured methylated *BCAT1* and *IKZF1* positivity rates by clinical findings.

Specimen Phenotype		Total	Sex	Median Age (min-max)	Bisulphite converted DNA Median (range), ng/mL	Single Marker Positivity n (%)		2-gene Positivity
						*BCAT1*	*IKZF1*	*n* (%)
**Healthy controls**		144	66F, 77M	56yrs (41–83)	8 (2–82)	5 (4%)	7 (5%)	11 (8%)
Collected by FMC		60	32F, 28M	61yrs (43–82)	6 (2–29)	2 (3%)	6 (10%)	7 (12%)
Collected by PGX		84	34F, 50M	55yrs (41–65)	11 (3–83)	3 (4%)	1 (1%)	4 (5%)
**Colorectal cancer**		74	39F, 36M	61yrs (32–86)	10 (2–731)	48 (65%)	50 (68%)	57 (77%)
Collected by FMC		25	8F, 17M	64yrs (47–86)	7 (2–731)	13 (52%)	15 (60%)	17 (68%)
Collected by PGX		49	30F, 19M	58yrs (32–78)	11 (2–155)	35 (71%)	35 (71%)	40 (82%)
**Stage**	Stage I	4	2F, 2M	62yrs (52–73)	7 (6–9)	1 (25%)	1 (25%)	2 (50%)
	Stage II	28	15F, 13M	58yrs (33–86)	38 (2–731)	18 (64%)	16 (57%)	19 (68%)
	Stage III	23	15F, 8M	60yrs (32–80)	38 (2–281)	15 (65%)	18 (78%)	20 (87%)
	Stage IV	8	2F, 6M	58yrs (40–72)	48 (10–154)	7 (88%)	8 (100%)	8 (100%)
	Stage Unknown	11	4F, 7M	68yrs (51–83)	10 (3–23)	7 (64%)	7 (64%)	8 (73%)

Mann-Whitney t-test (Gaussian, two-tailed) (combined marker results):

Positivity rates, Controls versus Cancers:

Complete dataset: Controls (n = 144, 11 positive) versus CRC cases (n = 74, 57 positive): p value <0.0001

PGX: Controls (n = 84, 4 positive) versus CRC cases (n = 49, 40 positive): p value <0.0001

FMC: Controls (n = 60, 7 positive) versus CRC cases (n = 25, 17 positive): p value <0.0001

### Detection of circulating methylated *BCAT1* and *IKZF1* DNA

**Fig 1:**
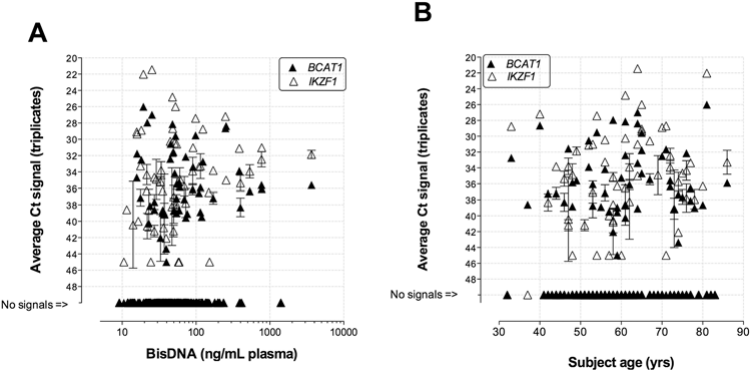
Methylation signal versus recovered DNA and subject age. Correlation plots of average Ct signals measured in the *BCAT1* (black triangles) and *IKZF1* (white triangles) methylation assays versus (A) mass of recovered bisulphite converted DNA per mL plasma or (B) age of subjects at the time of blood draw. Assay replicates with ‘no signal’ were assigned the arbitrary Ct value of ‘50’ for depiction purposes.

**Fig 2:**
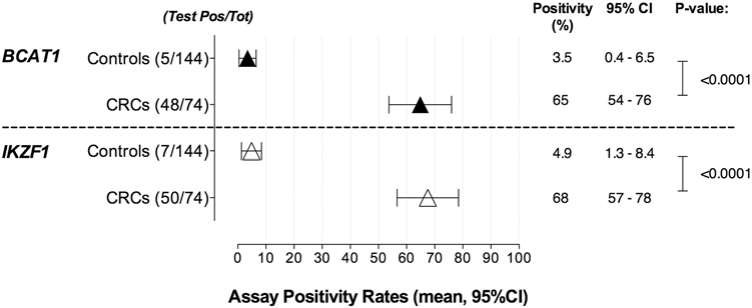
Detection of methylated *BCAT1* and *IKZF1* DNA in plasma specimens. *BCAT1* (top panel, black triangles) and *IKZF1* (bottom panel, white triangles) methylation specific assays were used to assess the appearance of circulating methylated *BCAT1* and *IKZF1* DNA in 218 plasma specimens including 144 healthy controls and 74 cancers. Mann-Whitney t-tests were performed on calculated positivity rates for each phenotypic class to derive the P-values. The calculated 95% confidence intervals (95% CI) are indicated.

The levels of methylated *BCAT1* and *IKZF1* DNA in bisulphite converted DNA recovered from the 218 plasma specimens were measured by triplicate analysis of an equivalent plasma volume of 0.5mL plasma per PCR well. Sixty-eight of the 218 plasma specimens were positive for either *BCAT1* and/or *IKZF1* methylation (Table 1 and S2 Table). No correlation was observed between assay positivity rates and yield of recovered bisulphite converted cfDNA, patient age (Fig 1) or gender (see S4 Fig). Statistically significant higher positivity rates were measured in CRC cases compared to controls for both methylation assays (Fig 2, p values <0.0001). The *BCAT1* methylation assay was positive in 53 of the 218 analysed specimens including 48 (65%) cancers and 5 (4%) controls. The *IKZF1* assay had a slightly higher positivity rate with 57 positive specimens including 50 (68%) CRC cases and 7 (5%) controls. Increasing positivity rates were observed with CRC stage for both markers as shown in Table 1. Lesion locations were known for 70 of the 74 cancer cases (S2 Table). There was no difference in the positivity rates measured in proximal versus distal cancers (proximal, n = 21, 16 positives (76%); distal, n = 49, 38 positives (78%), t-test p value: 0.9029).

### Methylation biomarker complementarity

**Fig 3:**
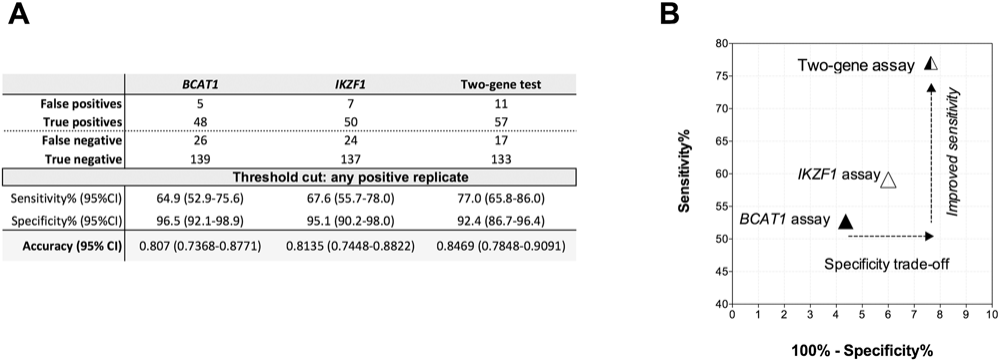
Discriminatory performance of one-gene versus two-gene testing. The sensitivity, specificity and discrimination accuracy values for the *BCAT1*, *IKZF1* and combined two-gene assays were calculated using the true and false positivity rates measured in 144 healthy control and 74 cancer cases (A) and subsequently plotted on Receiver Operator Characteristic (ROC) space plots (B). Single-gene assays: A plasma specimen was qualitatively called ‘positive’ if at least one PCR replicate resulted in a Ct signal. Two-gene assay: A specimen was qualitatively called ‘positive’ if at least one replicate in either the *BCAT1* or *IKZF1* assays produced a Ct value.

Sixty-eight specimens were positive in at least one of the two methylation assays, of which only 42 were positive for both methylation markers (41 CRC cases and 1 control). Fig 3A shows that a combined qualitative result based on at least one positive replicate in either the *BCAT1* or *IKZF1* methylation assay produced the highest classification accuracy (0.8469, 95%CI: 0.7848–0.9091) compared to either of the markers alone (*BCAT1*; 0.8070, 95%CI 0.7368–0.8771, *IKZF1*; 0.8135, 95%CI: 0.7448–0.8822). The 2-gene classifier model (“either or” rule) had an estimated sensitivity for CRC of 77% (95% CI: 65.8–86.0) with a specificity of 92.4% (95%CI: 86.7–96.4; Fig 3), thus improving the cancer detection rate by more than 10%, with less than a 4% decrease in specificity. The combined two-gene assay positivity for CRC stages I-IV was 50%, 68%, 87% and 100% respectively (Table 1).

### Levels of methylated *BCAT1* or *IKZF1* DNA versus disease severity

**Fig 4:**
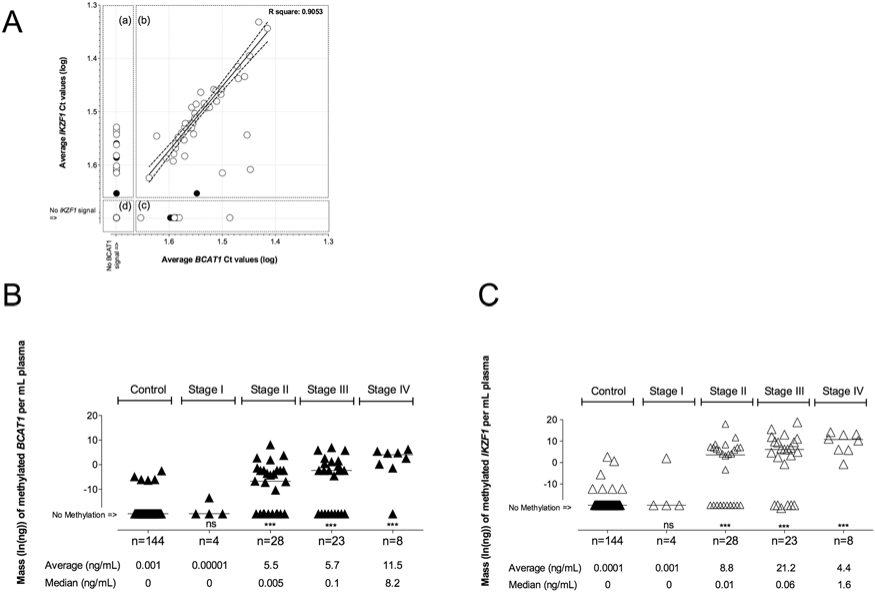
Mass of methylated *BCAT1* and *IKZF1* DNA in circulation. (A) Correlation plot of average Ct values (log(Ct)) measured by the *BCAT1* or *IKZF1* assays from 144 healthy controls (black circles) and 74 CRC (white circles). PCR replicates with no signal were assigned an arbitrary Ct value of 50 for depiction purposes. Square quadrants (a) to (d) represent: (a) 15 *BCAT1* negative but *IKZF1* positive cases; (b) 42 *BCAT1* and *IKZF1* positive cases including 41 cancer and 1 control; (c) 11 *BCAT1* positive but *IKZF1* negative cases; (d) 150 *BCAT1* and *IKZF1* negative cases. A linear regression analysis was performed on 38 of the 41 *BCAT1* and *IKZF1* positive CRC cases (quadrant b), omitting three CRC outliers. Broken diagonal lines indicate the 95% CI range. The calculated R square value is shown. The mass (ng) of methylated *BCAT1* (B) and *IKZF1* (C) DNA was calculated and plotted as mass of methylation per mL plasma drawn from 144 control cases, 4 Stage I, 28 Stage II, 23 Stage III, 8 Stage IV (the 8 CRC cases with unknown staging omitted). Average and median mass levels in the five phenotypic classes are indicated below the graphs. Mann-Whitney t-test performed on median values, ***: p value < 0.05, ns: non-significant, as compared with the 144 healthy control cases.

**Fig 5:**
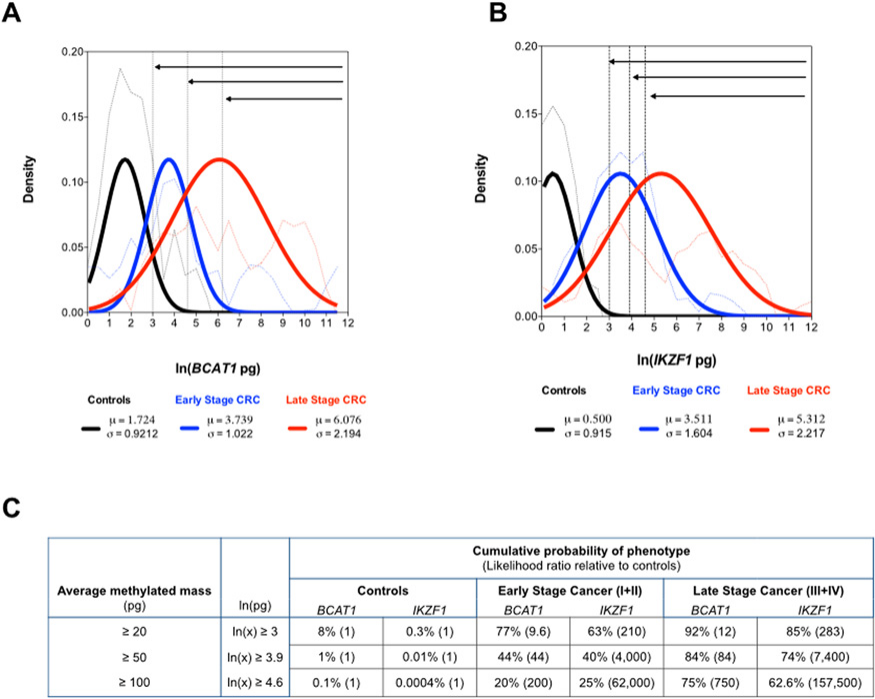
Estimation of class probabilities. The estimated average mass of (A) *BCAT1* and (B) *IKZF1* DNA in each of the triplicate assays containing DNA isolated from the equivalent of 0.5mL plasma from healthy controls (black lines, *BCAT1*: n = 5, *IKZF1*: n = 2*), early stage cancer (blue lines, Stage I+II, *BCAT1*: n = 19, *IKZF1*: n = 17) and late stage cancer (red lines, Stage III+IV, *BCAT1*: n = 22, *IKZF1*: n = 26) were used to calculate the empirical probability density plots, omitting ‘no signal’ results (broken lines). Solid lines: Population means (μ) and standard deviations (σ) were calculated assuming a normal distribution for each of the three phenotypic classifications. *Five of the seven positive *IKZF1* control cases had Ct signals within the last 5 cycles of PCR, therefore no mass estimation. (C) Cumulative probability of a plasma sample with *BCAT1* or *IKZF1* DNA methylation levels equal to or greater than 20-, 50- or 100pg methylated DNA coming from a patient with the indicated phenotypic classification; the mass thresholds were determined as the area under the curve (solid lines in panels A and B) for ln(pg) values greater than 3, 3.9 and 4.6, respectively (see dotted vertical lines and arrows) in Panels A and B. Likelihood ratios relative to healthy controls are shown in brackets.

Good correlation was observed in the cycle-threshold (Ct) values measured in specimens positive for both *BCAT1* and *IKZF1* DNA methylation (correlation co-efficiency R^2^ = 0.9053, Fig 4A). Each of the triplicate assays contained DNA isolated from the equivalent of 0.5 mL plasma. The average masses of methylated *BCAT1* or *IKZF1* DNA measured in CRC stages II, III and IV were significantly higher compared to the average mass of methylation measured in the limited number of controls that were positive (Fig 4B and 4C). The relationship between measured levels of circulating methylated *BCAT1* or *IKZF1* DNA in plasma and cancer stage was modeled to estimate the likelihood of cancer, given a methylated *BCAT1* or *IKZF1* DNA mass estimate per triplicate assay. Density plots were estimated (Fig 5A and 5B, broken lines) using only positive methylation masses for plasma of known phenotypes classified as healthy controls, early stage cancer (Stage I+II) and late stage cancer (Stage III+IV). The modeled density plots, assuming the observed methylation levels were drawn from a normal distribution (Fig 5A and 5B, solid lines), where used to estimate cumulative probabilities (Fig 5C) that a plasma sample from a known classification would yield an observed cancer-derived DNA methylation level equal to or greater than that corresponding to 20-, 50- or 100 pg. We determined that less than 8% of positive control plasma specimens are found to contain at least 20 pg of methylated *BCAT1* or *IKZF1* DNA, while 63–77% of early stage cancers had 20 pg or more. Thus the likelihood of a plasma specimen with ≥ 20 pg methylated *BCAT1* or *IKZF1* DNA to be an early stage cancer is approximately 9:1 and 210:1, respectively (early stage CRC: control). Further, a plasma specimen with 100 pg of methylated *IKZF1* DNA is approximately 2.5 times more likely to be drawn from a subject with late stage CRC (Stage III or IV) than a subject with early stage CRC (Stage I or II).

## Discussion

We have previously reported the development of two bisulphite conversion and methylation specific PCR assays used to detect colorectal neoplastic specific methylation in the gene loci *BCAT1* and *IKZF1* [9]. We optimized the *BCAT1* and *IKZF1* methylation qPCR assays for detection of methylation ratios as low as 0.1%, as such low levels of tumour-derived DNA have been reported in plasma specimens from patients with early stage CRC [4,15]. In this case-control study we demonstrate that methylated *BCAT1* and *IKZF1* DNA is detected more frequently in CRC cases (65% and 68%, respectively) than in colonoscopy-confirmed healthy controls (4% and 5%, respectively) and that the two methylation DNA markers are complementary (combined positivity of 77% and 7.6% in CRC and healthy controls, respectively).

A total of 41/74 cancer cases were positive for both methylated *BCAT1* and *IKZF1* DNA compared to only 1/144 control). Combining the results from the two assays resulted in a discriminatory accuracy of 0.8469 that was better than either assay alone (*BCAT1* assay: 0.807, *IKZF1* assay: 0.8135), Fig 3. Considering an either-or two-gene assay, more CRC cases were detected, with a correct segregation of 57/74 (77%) and only 11/144 (7.6%) healthy controls being misclassified. These two-gene data provide compelling, simple evidence that multiple biomarkers can improve sensitivity without major compromise of diagnostic utility in terms of lower specificity.

Detection of methylated *BCAT1* and *IKZF1* DNA in blood did not depend on age or gender of subject, or amount of recovered bisulphite converted cfDNA. A significant relationship was only observed to clinical phenotype. Both the frequency of detection and the amount of methylated *BCAT1* and/or *IKZF1* DNA released into blood increased with cancer stage. The latter was statistically significant in Stage II, III and IV cancers, with a higher methylation mass observed compared to the level measured in the few positive healthy control subjects (Fig 4). Similar observations have also been noted for the well-studied *SEPT9* methylation assay [16,17]. By fitting models of cancer classification (controls, early or late cancer), we show the potential of using the level of methylated *BCAT1* and/or *IKZF1* DNA in blood to estimate the probability of a positive result being due to the presence of cancer (Fig 5). Taken together, the data support a simple model where the ability to detect tissue-derived DNA markers in the circulatory system is a function of the extent of invasion by the lesion [18].

*BCAT1* and *IKZF1* are both involved in tumour growth and invasiveness [19,20]. A hypermethylated *BCAT1* locus has been demonstrated for several pathologies including CRC [21,22] and aberrant epigenetic regulation causes a ratio shift in three main *BCAT1* mRNA transcripts differing only in the first exon, which harbors alternative untranslated regions [20]. How the BCAT1 isoforms affect cell proliferation is unknown, but it is speculated that dysfunctional production of branched-chain amino acids may affect synthesis of macromolecules required for cell division, perhaps at the G1-to-S cell cycle control point [23].

The transcription factor, *IKZF1*, regulates numerous biological events and its role in lymphopoiesis and leukemic neoplasia has been extensively studied. A network of epigenetic and transcription factors regulate *IKZF1* gene activity [24] and emerging data imply that *IKZF1* is also crucial for proper regulation of proliferation and differentiation of cells of non-hematopoietic origin, including colon [25]. With the NuRD complex as the main interacting partner [26], Ikaros controls the transcriptional activity of a small set of genes [23,27], including *notch1* [28]. The Notch signaling pathway is critical for many cell-to-cell interactions, including the formation of invadopodia [29].

*SEPT9* is a negative regulator of pseudopodia formation [30], which is a key event that initiates cell dissemination and propulsion into surroundings tissue and stroma (recently reviewed by [31]). Thus, loss of *SEPT9* expression may play an active role in tumour invasion, where metastatic tumour cells through poorly understood mechanisms migrate to the vascular system [32]. We speculate whether *IKZF1* is perhaps an upstream regulator of *SEPT9* activity.

The Notch pathway plays a crucial role in the self-renewing process of colon crypt stem cells and their production of proliferating and transit-amplifying undifferentiated cell progenitors, which move up the crypt and into the villus where they further, differentiate [33,34]. We speculate if loss of *IKZF1* activity due to hypermethylation, leads to constitutively active Notch signaling which inhibits differentiation of crypt progenitor cells and results in a large increase in undifferentiated transient amplifying cells as previously described [35–37].

The increased positivity observed across stage I-IV cancers implies that detection of tumour-derived methylated DNA markers in blood may depend in large part on vascularity of colorectal neoplastic tissue and increased invasion in general [18,38]. If true, then a standardized sensitivity for detection of tumour-derived markers in circulation will be affected by the fraction of stage I cancers, as the majority of stage I cancers are unlikely to have any lymphatic or venous vessel invasion [18]. As such, blood-based assays are unlikely to detect a significant fraction of early stage cancers that can be detected by colonoscopy. This might explain the low sensitivity observed in a recent prospective evaluation of *SEPT9* in >7,900 asymptomatic participants where only 48% of 53 CRC cases (including twenty-two Stage I cancers) and 11% of adenomas were detected [17]. Our own dataset only included four Stage I cancers, and while two (50%) of these were detected, the sample numbers are presently too low to conclude whether the methylated *BCAT1*/*IKZF1* test will give good detection of Stage I cancers. Inclusion of vasculature-related variables (vascular invasion, vascular survival ability and tumour angiogenesis) appears to improve stratification of cancer patients [39]. Unfortunately, we could not assess whether detectability of methylated *BCAT1* and *IKZF1* DNA in blood was associated with appearance of vasculature-related features, as these variables were not recorded in the histological reports for the cancers included in the dataset herein.

## Conclusion

We report here for the first time a blood test where combining the detection of two methylation DNA biomarkers improves sensitivity for CRC without a substantial loss in specificity. The sensitivity for cancer was similar to published retrospective case control studies for the *SEPT9* methylation assay, although we report a higher specificity [8,11,16,40]. Further evaluation of this two-marker blood test across a broad range of clinical disorders and including pre-invasive neoplasia is now indicated.

## Supporting Information

S1 Fig *ACTB* PCR assay characteristics. Fourteen independent analyses of a 4-fold serial dilution of 5ng bisulphite converted blood DNA analysed in duplicates (n = 28). Good linearity (R2 square 0.9387) was measured from 5ng to 5 pg of DNA per PCR reaction. Graph data Ct values from each of the 28 replicates. No PCR signal was assigned an artificial Ct value of ‘50’. The total number of positive replicates, average Ct value (excluding Ct = 50) ± SD, and the 95%CI are shown for each pg DNA input point. Light grey band: Lower and upper 95%CI ranges of ACTB mean Ct values measured in 156 clinical plasma specimens collected by PGX, mean Ct value: 30.8, 95%CI range 30.19–30.57. Dark grey band: Lower and upper 9% CI ranges of ACTB mean CT values measured in 95 clinical plasma specimens from FMC (dark grey band, mean Ct value: 31.49, 95%CI range: 31.28–31.70).
(TIFF)

S2 Fig Analytical characteristics for the *BCAT1* and *IKZF1* assays. **(A):** Wild type DNA sequences (top sequence) and resulting bisulphite-converted fully methylated sequences (bottom sequence) detected and amplified by the *BCAT1* (top panel) and *IKZF1* (bottom panel) methylation specific PCR assays. Amplicon start coordinates are indicated (UCSC Genome browser GRCh38/hg38 version). Underline: methylated cytosines (marked with a “m”) residing in CpG sites. Red: unmethylated single cytosine residues converted to thymidines subsequent to bisulphite conversion and PCR. Arrows: bisulphite conversion and methylation specific forward and reverse primers (See S1 Table). Horizontal line in *BCAT1* diagram: position of a 5’-hydrolosis TaqMan probe. The quantitative ranges of the optimized methylation specific *BCAT1* (B) and *IKZF1* (C) assays were determined using a 7-point serial dilution of 5ng bisulphite converted fully methylated DNA in a background of bisulphite converted blood DNA (5ng total DNA per PCR well). Data graphs show each replicate from triplicate analysis of 14 (*BCAT1*) or 11 (*IKZF1*) independent standard curve analyses. Replicate positivities for each data point as well as resulting average Ct values ± SD and 95%CI are shown for both methylation assays.
(TIFF)

S3 Fig Mean concentration of recovered DNA. Yield (ng/mL plasma) was measured in plasma from colonoscopy-examined subjects from PGX (Panel A, n = 133: 84 controls, black circles; 49 CRCs, white circles) and FMC (Panel B, n = 85: 60 controls and 25 CRCs). No statistically significant difference was measured in the total DNA yields from the phenotypes collected from the same site. However, there were statistically significant differences in the phenotypic levels of total DNA recovered from PGX versus FMC (Mann-Whitney t-test).
(TIFF)

S4 Fig *BCAT1* and *IKZF1* assay positivity rates versus gender. Test pos/Tot: number of plasma samples tested positive for *BCAT1* (A) or *IKZF1* methylation (B) in 144 controls (including 66 females and 77 males) and 74 CRCs (including 39 females and 36 males). X-axis: Assay positivity rates and 95% confidence intervals are indicated for controls and CRCs. No statistically significant difference was observed between gender and *BCAT1/IKZF1* assay positivity (Mann-Whitney t-test).
(TIFF)

S1 Table PCR Details. (PDF)

S2 Table **Characteristics of the 218 patients included in the study UID:** Unique Identifier; **Source:** Participant recruitment site; **Age**: Patient age (years) at time of blood take; **Class:** colonic phenotype, CRC = colorectal cancer, Ctrl: controls; **TNM/Stage**: as defined by American Joint Committee on Cancer; **Lesion Site:** colonic segment involved. cec: ceacum, asc: ascending, hep: heptic flexure, tra: tranverse, spl: splenic flexure, des: descending, sig: sigmoid, rec: rectum; **Ave ACTB Ct:** Average cycle threshold value (duplicate), **STD ACTB:** Standard Deviation on average ACTB Ct values. **Ct_rep:** Cycle-threshold replicate values. **Estimated methylation:** Average yield (pg) measured in three replicates. #: Mass values cannot be accurately extrapolated from amplification occurring in the last five cycles of the 50-cycle IKZF1 SYBR-green PCR, which in such case are assigned a Ct value of 45.
(XLS)
