# Supplementary figures and images for: A Two-Gene Blood Test for Methylated DNA Sensitive for Colorectal Cancer
Source: PLoS One. 2015 Apr 30;10(4):e0125041. doi: 10.1371/journal.pone.0125041 (PMC4416022; doi:10.1371/journal.pone.0125041)

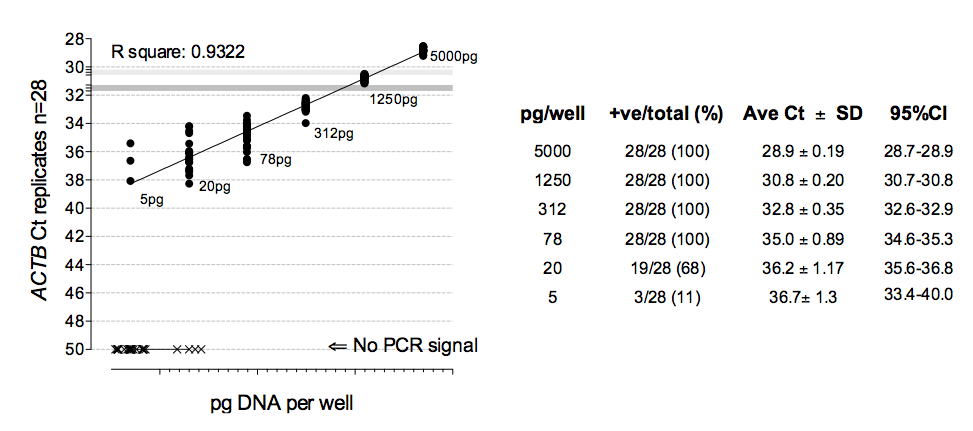

Supplement: S1 Fig — Fourteen independent analyses of a 4-fold serial dilution of 5ng bisulphite converted blood DNA analysed in duplicates (n = 28). Good linearity (R2 square 0.9387) was measured from 5ng to 5 pg of DNA per PCR reaction. Graph data Ct values from each of the 28 replicates. No PCR signal was assigned an artificial Ct value of ‘50’. The total number of positive replicates, average Ct value (excluding Ct = 50) ± SD, and the 95%CI are shown for each pg DNA input point. Light grey band: Lower and upper 95%CI ranges of ACTB mean Ct values measured in 156 clinical plasma specimens collected by PGX, mean Ct value: 30.8, 95%CI range 30.19–30.57. Dark grey band: Lower and upper 9% CI ranges of ACTB mean CT values measured in 95 clinical plasma specimens from FMC (dark grey band, mean Ct value: 31.49, 95%CI range: 31.28–31.70). (TIFF) [file pone.0125041.s001.tiff]

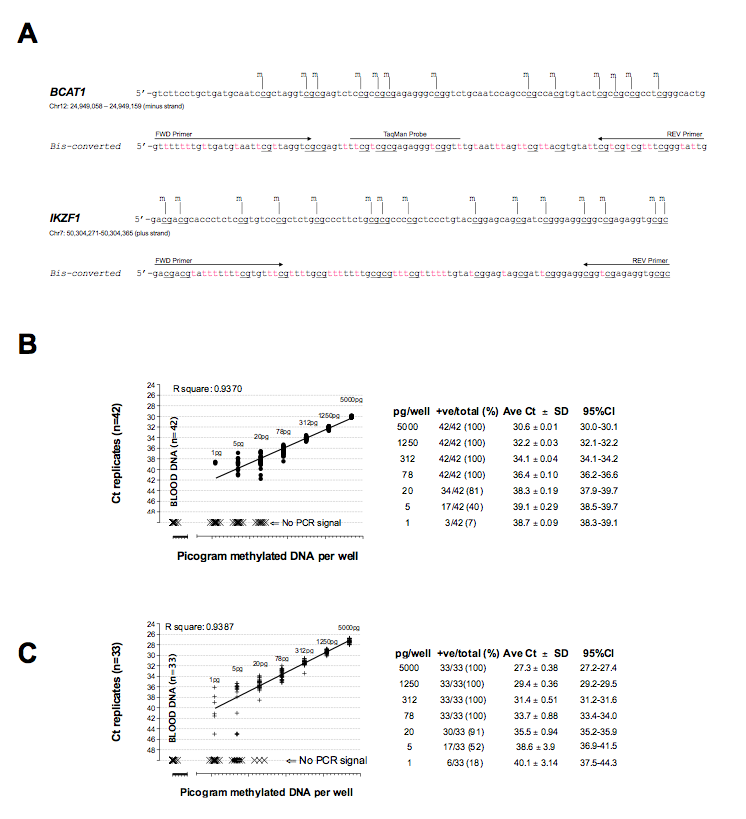

Supplement: S2 Fig — (A): Wild type DNA sequences (top sequence) and resulting bisulphite-converted fully methylated sequences (bottom sequence) detected and amplified by the BCAT1 (top panel) and IKZF1 (bottom panel) methylation specific PCR assays. Amplicon start coordinates are indicated (UCSC Genome browser GRCh38/hg38 version). Underline: methylated cytosines (marked with a “m”) residing in CpG sites. Red: unmethylated single cytosine residues converted to thymidines subsequent to bisulphite conversion and PCR. Arrows: bisulphite conversion and methylation specific forward and reverse primers (See S1 Table). Horizontal line in BCAT1 diagram: position of a 5’-hydrolosis TaqMan probe. The quantitative ranges of the optimized methylation specific BCAT1 (B) and IKZF1 (C) assays were determined using a 7-point serial dilution of 5ng bisulphite converted fully methylated DNA in a background of bisulphite converted blood DNA (5ng total DNA per PCR well). Data graphs show each replicate from triplicate analysis of 14 (BCAT1) or 11 (IKZF1) independent standard curve analyses. Replicate positivities for each data point as well as resulting average Ct values ± SD and 95%CI are shown for both methylation assays. (TIFF) [file pone.0125041.s002.tiff]

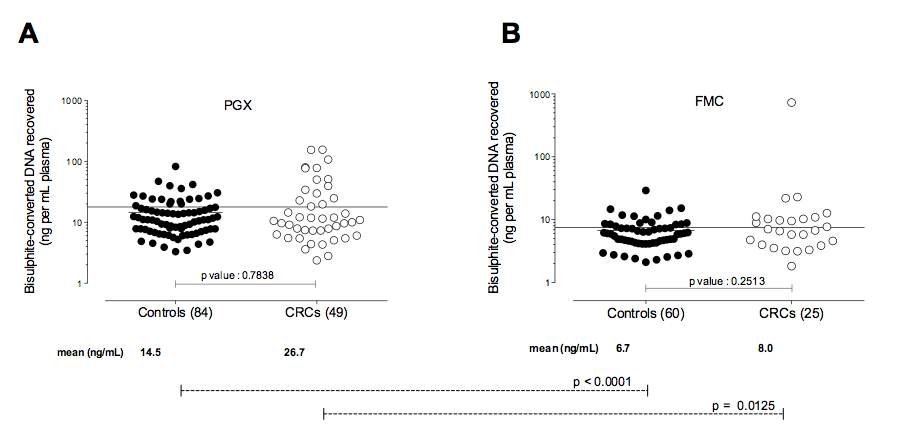

Supplement: S3 Fig — Yield (ng/mL plasma) was measured in plasma from colonoscopy-examined subjects from PGX (Panel A, n = 133: 84 controls, black circles; 49 CRCs, white circles) and FMC (Panel B, n = 85: 60 controls and 25 CRCs). No statistically significant difference was measured in the total DNA yields from the phenotypes collected from the same site. However, there were statistically significant differences in the phenotypic levels of total DNA recovered from PGX versus FMC (Mann-Whitney t-test). (TIFF) [file pone.0125041.s003.tiff]

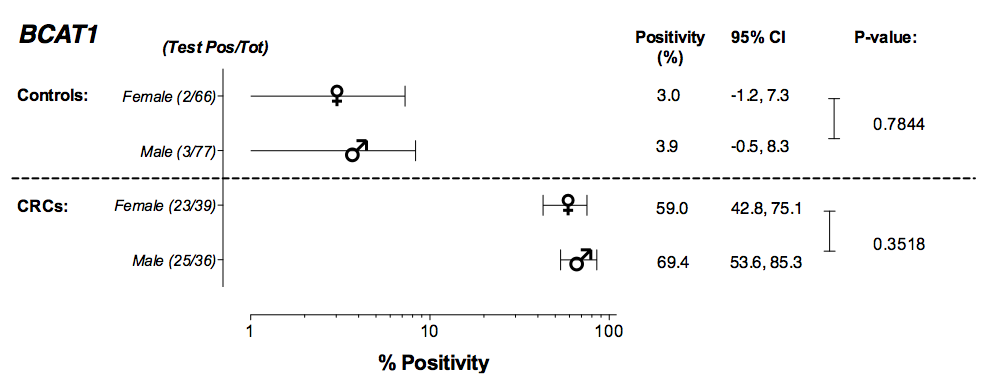

Supplement: S4 Fig — Test pos/Tot: number of plasma samples tested positive for BCAT1 (A) or IKZF1 methylation (B) in 144 controls (including 66 females and 77 males) and 74 CRCs (including 39 females and 36 males). X-axis: Assay positivity rates and 95% confidence intervals are indicated for controls and CRCs. No statistically significant difference was observed between gender and BCAT1/IKZF1 assay positivity (Mann-Whitney t-test). (TIFF) [file pone.0125041.s004.tiff]
