# Supplementary material for: A Two-Gene Blood Test for Methylated DNA Sensitive for Colorectal Cancer
Source: PLoS One. 2015 Apr 30;10(4):e0125041. doi: 10.1371/journal.pone.0125041 (PMC4416022; doi:10.1371/journal.pone.0125041)
Supplement: S1 Table — (PDF) [file pone.0125041.s005.pdf]

S1 Table: PCR details

Gene	Oligonucleotides (Sequences given from 5'-end to 3'-end)	PCR component concentrations[3] (in 15μL)	PCR cycling conditions[4]
ACTB	FWD[2]: GTGATGGAGGAGGTTTAGTAAGTT<br>REV[2]: AATTACAAAAACCACAACCTAATAAA<br>Pr[2]: [6FAM]ACCACCACCCAACACACAATAACAAACACA[BHQ1]	5μL 1:5 bis-DNA[1], 3mM $MgCl_2$, 200μM dNTPs, 900nM FWD, 900nM REV, 100nM Pr, 0.05U/μL Platinum Taq DNA polymerase, 1X Platinum Taq Buffer	1x [95°C/2min],<br>50x [95°C/10s; 60°C/50s]<br>1x [40°C/10s]
BCAT1	FWD: GTTTTTTTGTTGATGTAATTCGTTAGGTC<br>REV: CAATACCCGAAACGACGACG<br>Pr: [HEX]TTCGTCGCGAGAGGGTCGGTT[BHQ1]	5μL bis-DNA, 4mM $MgCl_2$, 200μM dNTPs, 200nM FWD, 200nM REV, 100nM Pr, 0.033U/μL Platinum Taq DNA polymerase, 1X Platinum Taq Buffer	1x [95°C/2min],<br>50x [95°C/15s; 62°C/30s; 72°C/30s]<br>1x [40°C/10s]
IKZF1	FWD: GACGACGTATTTTTTTCGTGTTTC<br>REV: GCGCACCTCTCGACCG	5μL bis-DNA, 200nM FWD, 200nM REV, 1:100,000 SYBR Green, 1xGoTaq HotStart Mastermix, 1mM additional $MgCl_2$	1x [95°C/2min],<br>50x [95°C/15s; 62°C/30s; 72°C/30s]<br>1x melt analysis [5]<br>1x [40°C/10s]

[1]bis-DNA: bisulphite-converted DNA isolated from 4mL plasma; [2]FWD: forward primer, REV: reverse primer, Pr: dual-labelled hydrolysis probe; [3] $MgCl_2$ (Invitrogen, New York, United States), dNTPs (Invitrogen, New York, United States) FWD/REV/Pr (Sigma Aldrich, Sydney, Australia), Platinum Taq DNA polymerase (Life Technologies, New York, United States), 2X GoTaq Hot Start Buffer w/ $MgCl_2$ (Promega, Wisconsin, United States), SYBR Green, Molecular Grade (Molecular Probes, Oregon, United States); [4] Roche LightCycler 480 Model. [5] Melt analysis was 1 cycle of 95°C/10s; 65°C/60s; ramp to 97°C at 0.11°C/sec with continuous acquisition.
